# Bioequivalence and safety assessment of sorafenib tosylate tablets in healthy Chinese subjects under fasting conditions

**DOI:** 10.3389/fphar.2025.1470095

**Published:** 2025-04-14

**Authors:** Zhaoyu Wang, Hong Peng, Yun Zhang, Xijing Chen, Jiye A

**Affiliations:** ^1^ Clinical Pharmacology Laboratory, School of Basic Medicine and Clinical Pharmacy, China Pharmaceutical University, Nanjing, China; ^2^ Jiangsu Provincial Key Laboratory of Drug Metabolism and Pharmacokinetics, China Pharmaceutical University, Nanjing, China

**Keywords:** sorafenib, bioequivalence, pharmacokinetic, UPLC-MS/MS, safety

## Abstract

**Objective:**

This study aimed to assess the bioequivalence and safety of two formulations of sorafenib in healthy Chinese subjects under fasting conditions.

**Methods:**

A single-center, randomized, open, single-dose, two-formulation, four-period, crossover study was performed in 36 healthy Chinese subjects under fasting conditions. Blood samples were collected within 120 h after administration. The plasma concentrations of sorafenib were analyzed by a validated UPLC-MS/MS method, and pharmacokinetic parameters were analyzed using a non-compartmental method. Safety was assessed on the basis of the occurrence of adverse events and laboratory findings throughout the study period.

**Results:**

The GMR point estimators of C_max_, AUC_0-t_, and AUC_0- ∞_ for the two formulations were 88.97%, 81.67%, and 83.66%, respectively, which were within the bioequivalence criterion range of 80%–125%. The upper limits of the one-sided 95% confidence intervals of C_max_, AUC_0-t_, and AUC_0- ∞_ after logarithmic transformation were −0.05, −0.04 and −0.03, respectively, which were less than 0. The difference in T_max_ between these two formulations was not statistically significant according to the Wilcoxon signed-rank test (P = 0.3650 > 0.05). Therefore, the bioequivalence between the two formulations was established under fasting conditions. All adverse events were mild and transient.

**Conclusion:**

The T formulation was bioequivalent and showed a similar safety profile to the R formulation Nexavar^®^ (Bayer AG) in healthy Chinese subjects under fasting conditions.

**Clinical trial registration:**

http://www.chinadrugtrials.org.cn/index.html, Identifier CTR20233578.

## 1 Introduction

Sorafenib (BAY 43-9006, Nexavar^®^) is a novel lipophilic small molecule with a biaryl urea structure that belongs to the Biopharmaceutics Classification System (BCS) II because of its low solubility and high membrane permeability (Cui et al., 2022; Ranieri et al., 2012; Ramakrishnan et al., 2012; Huh et al., 2021). After oral administration, sorafenib effectively inhibited tumor cell proliferation by targeting rapidly accelerated fibrosarcoma serine-threonine kinases (Raf). Additionally, it exhibits significant inhibitory activity against receptor tyrosine kinases such as vascular endothelial growth factor receptor (VEGFR) and platelet-derived growth factor receptor β, which help reduce tumor angiogenesis (Wilhelm et al., 2004; Wilhelm et al., 2008; Liu et al., 2006). Sorafenib tablets (200 mg) were approved by the Food and Drug Administration (FDA) in 2005 for the treatment of patients with advanced renal cell carcinoma (RCC) (Kane et al., 2006). It is also the first drug approved as a first-line treatment for advanced hepatocellular carcinoma (HCC) as a first-line treatment (Cui et al., 2022).

The pharmacokinetics of sorafenib varies greatly among individuals after a single oral dose. However, food intake does not significantly affect metabolic profiles (Strumberg et al., 2005). Sorafenib reaches its peak concentration approximately 3 h after oral administration, and its mean elimination half-life is 25–48 h (Kane et al., 2006). Sorafenib is mainly metabolized in the liver via two pathways: phase I oxidative metabolism through CYP3A4, and phase II glucuronidation via UGT1A9 (Keating and Santoro, 2009). After oral administration of a 100 mg solution formulation, 77% of the dose is excreted in the feces and 19% in the urine as glucuronidated metabolites. The prototype drug is mostly excreted in feces at 51% of the dose (Kane et al., 2006).

Common adverse drug reactions associated with sorafenib administration include gastrointestinal issues such as diarrhea, nausea, anorexia, and dermatological issues such as hand-foot syndrome (HFS), skin dryness, rash, pruritus, alopecia, stomatitis, and fatigue (Clark et al., 2005; Awada et al., 2005; Moore et al., 2005; Strumberg et al., 2005). Most patients with advanced refractory solid tumors experience mild to moderate severity of these adverse events, indicating that sorafenib is generally well-tolerated and safe (Strumberg et al., 2007).

Currently, studies on the pharmacokinetics of sorafenib in healthy Chinese subjects are limited, necessitating further investigation. This trial aimed to evaluate the pharmacokinetics, bioequivalence, and safety of two sorafenib tosylate tablets obtained from different manufacturers (Renhexidelong Pharmaceutical Co. Ltd., and Bayer AG, Germany) after single-dose administration under fasting conditions in healthy Chinese adults.

## 2 Methods

### 2.1 Study design

This study adhered to the principles of the Declaration of Helsinki (World Medical Association, 2013), Good Clinical Practice (GCP) (NMPA, National Medical Products Administration, National Health Commission of the People’s Republic of China, 2020), and the guidelines of the National Medical Products Administration (NMPA) (NMPA, The National Medical Products Administration of China, Center for Drug Evaluation, 2019) of China. The trial was registered with the number CTR20233578, conducted at the Phase I Clinical Trial Center of Zhonghui Cardiovascular Disease Hospital, Henan (Zhengzhou), from 1 December 2023, to 4 February 2024, and approved by the Institutional Ethics Committee before initiation.

**FIGURE 1:**
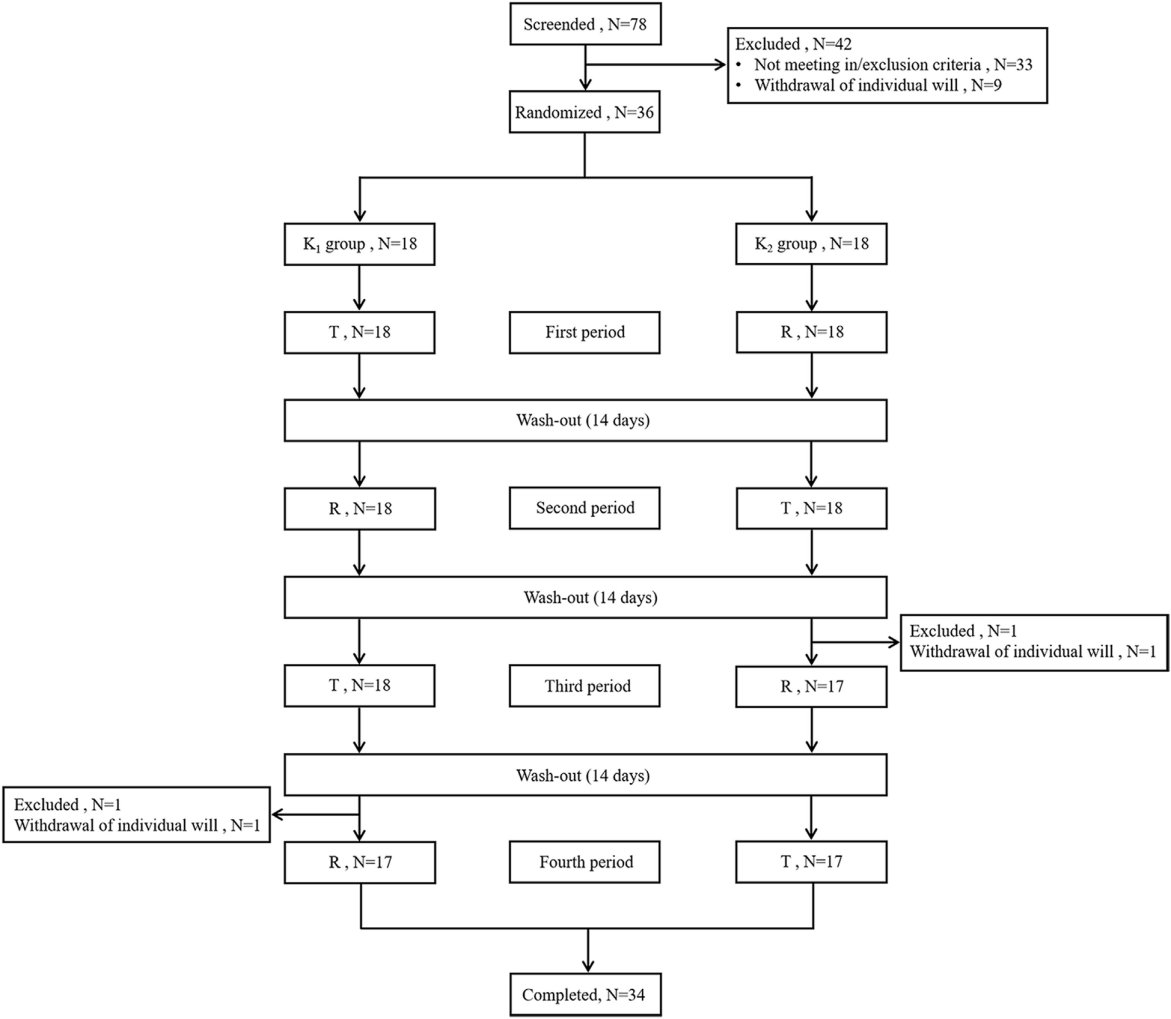
Flow chart of the study. Abbreviations: T, test formulation; R, reference formulation; N, number of subjects.

This trial involved a single-center, randomized, open, single oral administration, two-formulation, four-period crossover study and featured a screening period, four treatment periods, a washout period of 14 days after each treatment period, and a follow-up period following the last treatment (Figure 1). A total of 36 healthy male and female Chinese subjects were included in the study and randomly assigned to either the K_1_ group (TRTR) or K_2_ group (RTRT) with equal sex ratios using SAS software (v9.4). The subjects were admitted to the Phase I Clinical Research Center the day before dosing and fasted overnight (10 h). All subjects received a single oral administration of 0.2 g of sorafenib tosylate tablets (T formulation,0.2 g, lot SRF1230602, Renhe Xidelong Pharmaceuticals Co., Ltd.) or Nexavar^®^(R formulation, 0.2 g, lot: BXJX3D1, Bayer AG) on the day of dosing with 240 mL of water. The participants were not allowed to drink additional water for 1 h before or after treatment. and forbidden from walking or eating for 4 h after administration.

### 2.2 Subjects

All participants received group and individual counseling sessions with the investigator to understand the trial process and risks, and signed informed consent forms prior to participation.The inclusion criteria are healthy male and female subjects aged 18 years and above; male subjects weighing ≥50 kg and female subjects weighing ≥45 kg with a body mass index (BMI) of 19.0–26.0 kg/m^2^; full understanding of the experimental procedure and risks; and no birth plan from the beginning of the experiment to 3 months after its completion.

The exclusion criteria were participation in any other clinical trial of a drug within the past 3 months, presence of any clinically relevant medical condition or history, inability to tolerate venipuncture, blood or needle sickness, blood donation or blood loss ≥400 mL within the past 3 months, abnormal and clinically significant physical examination, electrocardiogram, vital signs, laboratory tests, and hypersensitivity to the study drug. Pregnant and lactating women were excluded from this study.

### 2.3 Collection and preservation of blood samples

Blood samples (N = 22) were collected from before drug administration to120 h after administration for each subject at the following time points: 0 h (immediately before dosing) and 0.5, 1, 1.5, 2, 2.5, 3, 3.5, 4, 4.5, 5, 6, 8, 9, 10, 11, 12, 24, 48, 72, 96 and 120 h after administration. A total of 4 mL of whole blood was collected into EDTA-K_2_ vacuum blood collection tubes. The blood samples were centrifuged at 3,000 g for about 5 min at 2°C–8°C within 1 hour, All plasma samples were frozen at −70°C (−60 to −90°C) within 2 h of blood collection.

### 2.4 Analytical determinations

A methodologically validated UPLC-MS/MS method was used to determine the plasma concentration of sorafenib. Ultra-performance liquid chromatography was performed using the LC-40D system (Shimadzu, Japan), while the mass spectrometer was the AB Sciex QTRAP 6500+ (Applied Biosystems, United States). The data acquisition software was Analyst 1.7.3 (Applied Biosystems, United States).

Plasma samples containing the drug were processed using a protein precipitation method. The internal standards were sorafenib-d3 (QCSRM, lot number: 24213; purity: 97.51%) and sorafenib toluenesulfonate (China Academy of Food and Drug Control, lot number: 420138-202201; content: 99.7%). The internal standard precipitant was Sorafenib-d3 diluted with acetonitrile at a concentration of 20.00 ng/mL. The drug-containing plasma was thawed at room temperature and then precipitated by a 10-fold dilution with the internal standard precipitant. The mixture was vortexed for 5 min, centrifuged at 15,000 rpm and 4°C for 10 min, and the supernatant was used for UPLC-MS/MS analysis. The plasma concentration range of the standard curve for this analytical method was 20–3,000 ng/mL,and the LLOQ, LQC, MQC and HQC of the substances to be measured were 20.00, 60.00, 300.0 and 2400 ng/mL, respectively.The intra-lot accuracy deviations of all QC samples ranged from −5.5% to 5.2% with a precision maximum (%CV) of 1.25%; the inter-lot accuracy deviations ranged from −5.5% to 5.9% with a %CV of 1.72%.

Chromatographic separation of the samples was performed on an ACQUITY UPLC^®^BEH C18 column (1.7 μm, 50 × 2.1 mm). Gradient elution of sorafenib was performed using 2 mM ammonium formate-water (A): acetonitrile (B) as the mobile phase at a flow rate of 0.5 mL/min and column temperature of 40°C. Positive ionization mode was used to detect sorafenib in the plasma samples. The mass-to-charge ratios (m/z) of sorafenib and sorafenib-d3 were 465.2→252.2 and 468.1→255.2 respectively.

### 2.5 Pharmacokinetic and statistical analysis

The pharmacokinetic parameters of sorafenib were calculated using a non-compartmental method. C_max_ and T_max_ are the peak concentration and time, respectively, which were obtained directly from the measured blood concentration-time (C-T) curves. AUC_0-t_ is the area under the blood concentration-time curve from drug administration to the last point of blood sampling, calculated using the linear trapezoidal rule. AUC_0- ∞_ is the area under the curve from drug administration to extrapolation to infinity. t_1/2_ is the elimination half-life, and these parameters and the mean sorafenib blood concentration-time plot were obtained using Phoenix WinNonlin software (V 8.3).

Statistical analysis was performed using the SAS software (V 9.4). A descriptive analysis of the subjects’ baseline characteristics, such as sex, age, height, weight, and body mass index, as well as pharmacokinetic concentration data, was performed. Means, standard deviations, maximum and minimum values, medians, and first and third quartiles (Q1,Q3) were calculated for continuous variables, while frequencies and percentages were calculated for categorical variables. The main pharmacokinetic parameters (C_max_, AUC_0-t_, and AUC_0- ∞ _) were log-transformed and analyzed by analysis of variance (ANOVA) (NMPA, The National Medical Products Administration of China, Center for Drug Evaluation, 2019). The sequences, drugs, and periods were considered fixed effects, while subjects were treated as random effects in the ANOVA model. A two-sided t-test was conducted to evaluate the bioequivalence of the two formulations, and a non-parametric test of crossover design (Wilcoxon test) was used to analyze the statistical significance of the differences in the time to peak between the preparations. The T formulation was considered equivalent to the R formulation if the 90% confidence interval of the log-transformed geometric mean ratio of AUC_0-t_, AUC_0- ∞ _, and C_max_ fell within the range of 80%–125%.

### 2.6 Safety evaluations

Safety analyses were performed for subjects who had received at least one dose of the test drug. The safety of sorafenib tosylate tablets was assessed based on adverse event reports, vital signs (including seated blood pressure, pulse, and temperature), physical examinations, 12-lead electrocardiograms, and clinical laboratory tests (routine blood, urine, blood biochemistry, coagulation, and virologic testing). Vital signs were measured and recorded respectively within 1 hour before and 2, 4, 6, 8, 12, 24, 48, 72, 96, and 120 h after dosing during each period. In addition, the subjects underwent clinical laboratory testing, physical examination, and 12-lead electrocardiography at screening and before withdrawal from the study. All post-dose adverse events were monitored throughout the trial and assessed according to the Common Terminology Criteria for the Evaluation of Adverse Events (CTCAE V 5.0). All adverse events were coded using the Preferred Terminology of the International Medical Terminology Dictionary (MedDRA V 26.0) and categorized and summarized according to system organ classification and preferred terminology.

## 3 Results

### 3.1 Subject characteristics

A total of 78 healthy adult subjects were screened in this study, of which 42 failed screening and 36 (29 males and 7 females) were successfully enrolled and randomly assigned to either the K_1_ (TRTR) or K_2_ (RTRT) group, which had 18 participants each. All participants received the administered drug. A total of 34 subjects completed the trial, and two subjects withdrew from the study early willingly (one in the third period and the other in the fourth period). The demographic information and baseline characteristics of the K_1_ and K_2_ groups were group-balanced. The demographic data for all subjects is summarized in Table 1, and the flow chart of the study is shown in Figure 1.

**TABLE 1:** The demographic and baseline characteristics of subjects in the clinical trials.

Variable	K_1_ group (TRTR,N = 18)	K_2_ group (RTRT,N = 18)	Total (N = 36)
Gender [n (%)]			
Male	14 (77.8)	15 (83.3)	29 (80.6)
Female	4 (22.2)	3 (16.7)	7 (19.4)
Age (year)			
Mean (SD)	30.94 (8.69)	28.67 (7.50)	29.81 (8.08)
Min,Max	20, 44	18, 49	18, 49
Race [n (%)]			
Han	18 (100)	18 (100)	36 (100)
Other	0 (0.0)	0 (0.0)	0 (0.0)
Height (cm)			
Mean (SD)	168.61 (7.17)	171.06 (5.40)	169.83 (6.38)
Min,Max	155, 179	163, 182	155, 182
Weight (kg)			
Mean (SD)	65.94 (8.70)	67.46 (7.64)	66.70 (8.11)
Min,Max	48.1, 79.8	52.5, 79.3	48.1, 79.8
BMI (kg/m^2^)			
Mean (SD)	23.12 (2.03)	23.04 (2.22)	23.08 (2.10)
Min,Max	19.8, 25.6	19.1, 25.9	19.1, 25.9

Abbreviations: K_1_, K_1_ group (TRTR); K_2_, K_2_ group (RTRT); N, number of subjects; T, test formulation; R, reference formulation; BMI, body mass index; SD, Standard Deviation.

### 3.2 Pharmacokinetics of sorafenib

**FIGURE 2:**
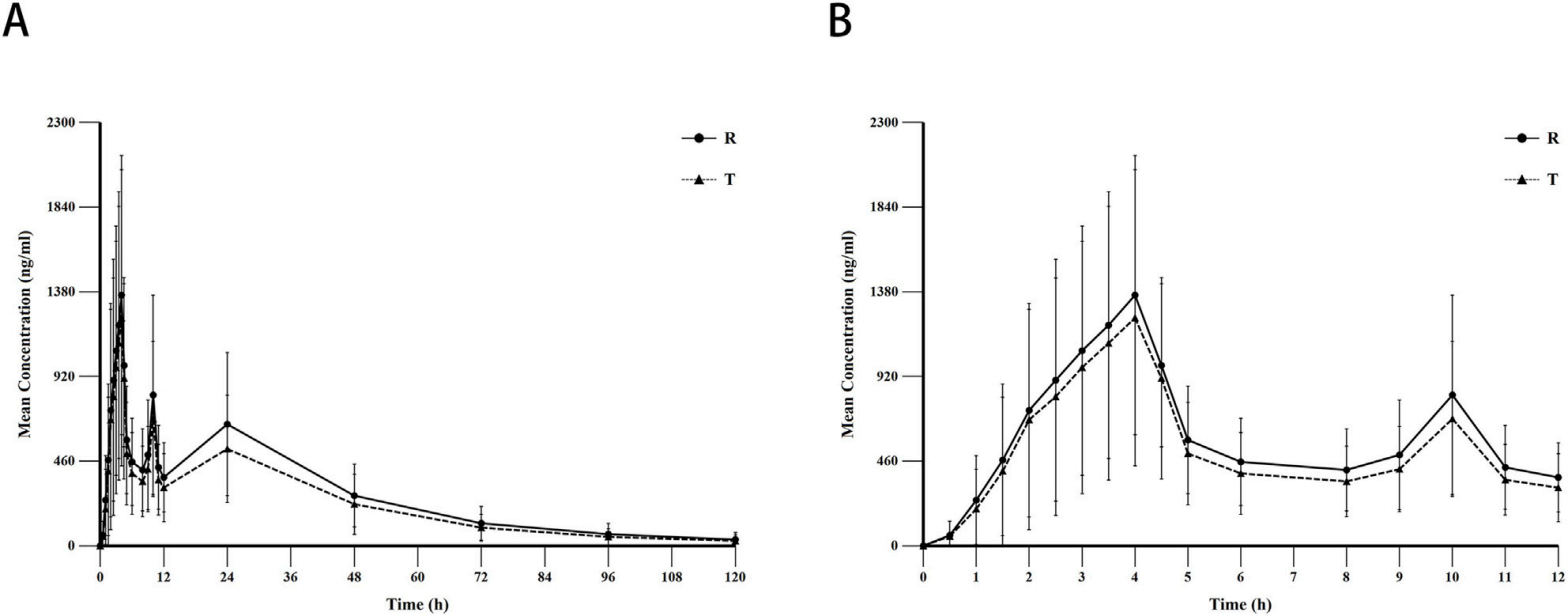
The mean blood concentration-time curves for sorafenib after oral administration of the test (N = 71) and reference formulation (N = 70) under fasting conditions. Notes: **(A)**, linear scale for 120 h; **(B)**, linear scale for first 8 h. Abbreviations: T, test formulation; R, reference formulation.

Data from the blood concentration studies of all 36 subjects were included in the pharmacokinetic analysis. The mean blood concentration-time curves for sorafenib after oral administration of the T formulation and R formulation under fasting conditions are shown in Figure 2. The pharmacokinetic parameters for the T and R formulations are summarized in Table 2.

**TABLE 2:** Pharmacokinetic parameters of sorafenib after administration of T and R formulations under fasted conditions.

PK parameters	T Formulation (N = 71)	R Formulation (N = 70)
C_max_ (ng/mL)	1,306.7 ± 806.20 (61.7%)	1,451.2 ± 753.73 (51.9%)
T_max_ (h)	4.0 (3,24)	4.0 (3,10)
AUC_0-t_ (ng·h/mL)	26,759.7 ± 15,437.47 (57.7%)	32,512.8 ± 18,125.10 (55.7%)
AUC_0- ∞ _ (ng·h/mL)	28,597.5 ± 15,783.78 (55.2%)	34,410.6 ± 18,933.27 (55.0%)
t_1/2_ (h)	25.8 ± 11.58 (45.0%)	24.3 ± 9.71 (39.9%)
λ_z_ (1/h)	0.0 ± 0.01 (31.7%)	0.0 ± 0.01 (28.5%)

Note: Data are shown as mean ± SD (CV%), except that T_max_ (h) shown as median (min, max). Abbreviations: T, test formulation; R, reference formulation; N, number of subjects; SD, Standard Deviation; C_max_, maximum observed plasma concentration; T_max_, time to maximum observed plasma concentration; AUC_0-t_, area under the curve from time 0 to the time of the last quantifiable concentration; AUC_0- ∞ _, area under the curve from time 0 extrapolated to infinity; t_1/2_, terminal elimination half-life; λ_z_,terminal elimination rate constant.

The mean ± SD (CV%) of the C_max_ values for the T and R formulations were 1,306.7 ± 806.20 (61.7%) ng/mL and 1,451.2 ± 753.73 (51.9%) ng/mL, respectively; the AUC_0-t_ values were 26,759.7 ± 15,437.47 (57.7%) ng·h/mL and 32,512.8 ± 18,125.10 (55.7%) ng·h/mL, respectively; the AUC_0- ∞_ values were 28,597.5 ± 15,783.78 (55.2%) ng·h/mL and 34,410.6 ± 18,933.27 (55.0%) ng·h/mL, respectively. The median T_max_ values for two formulations were both 4.0 h. The mean ± SD (CV%) of the λz values for the T and R formulations were 0.0 ± 0.01 (31.7%) 1/h and 0.0 ± 0.01 (28.5%) 1/h, respectively, and the t_1/2_ values were 25.8 ± 11.58 (45.0%) h and 24.3 ± 9.71 (39.9%) h, respectively.The pharmacokinetic profile of sorafenib generally exhibits enterohepatic reabsorption characterized by multiple peaks, a long clearance half-life, and slow terminal elimination.

### 3.3 Bioequivalence analysis

In this study, the intra-individual standard deviation (S_WR_) of C_max_, AUC_0-t_, and AUC_0- ∞_ of the T formulation were 0.40, 0.43, 0.42, respectively, which were all ≥0.294, so the bioequivalence evaluation was carried out by the reference-scaled average bioequivalence (RSABE) approach.The GMR point estimators of C_max_, AUC_0-t_, and AUC_0- ∞_ were 88.97%, 81.67%, and 83.66%, respectively, and fell within the bioequivalence standard range of 80%–125%. Meanwhile, the upper limits of the one-sided 95% confidence intervals for C_max_, AUC_0-t_, and AUC_0- ∞_ after the natural logarithmic transformation were −0.05, −0.04, and −0.03, respectively, which were all less than 0. In addition, the differences between T_max_ of the T and R formulations were not statistically significant, as shown by the Wilcoxon signed-rank test (p = 0.3650 > 0.05). These results indicate that the T and R formulations are bioequivalent under fasting conditions. The results of the bioequivalence evaluation between the T and R formulations under the fasting status are shown in Table 3.

**TABLE 3:** Analysis of bioequivalence for plasma pharmacokinetic parameters of sorafenib under fasted conditions.

PK parameters	GM		ABE		RSABE			
	T (N = 71)	R (N = 70)	T/R (%)	90%CI (Lower, Upper)	CV_WR_ (%)	CV_WT_ (%)	GMR point estimators [80.00%,125.00%]	Critical Bound [≤0]
C_max_ (ng/mL)	1,083.97	1,250.43	86.69	(75.89,99.02)	41.93	39.82	88.97	−0.05
AUC_0-t_ (ng·h/mL)	22,213.80	27,570.57	80.57	(71.78,90.44)	45.64	38.14	81.67	−0.03
AUC_0- ∞ _ (ng·h/mL)	24,444.11	29,448.84	83.01	(74.64,92.31)	43.75	35.16	83.66	−0.04

Notes: Within-subject coefficient of variation (CV%) was calculated using the exact formula:CV% = 100×sqrt (exp (S_wr_^2)-1), where S_wr_^2 is the within-subject variance estimated from the linear mixed-effects model. Abbreviations: PK, Pharmacokinetics; GM, geometric mean; ABE, average bioequivalence; RSABE, reference-scaled average bioequivalence; T, test formulation; R, reference formulation; CI, confidence intervals; CV_wr_, within-subject coefficient of variation (reference); CV_wt_, within-subject coefficient of variation (test); GMR, geometric mean ratios; C_max_, maximum observed plasma concentration; AUC_0-t_, area under the curve from time 0 to the time of the last quantifiable concentration; AUC_0- ∞ _, area under the curve from time 0 extrapolated to infinity.

### 3.4 Safety

A total of 7 instances of TEAE were reported in 5 out of the 36 subjects after administering the T formulation. All 7 instances were possibly related to the study drug, resulting in a TEAE incidence rate of 13.89% for the T formulation. In contrast, participants experienced a total of 4 instances of TEAE in 4 subjects after receiving the R formulation with only 1 instance being possibly related to the study drug, and the remaining 3 instances being possibly unrelated to the T formulation, which resulted in a TEAE incidence rate of 11.11% for the R formulation. Table 4 displays the status and comparison of adverse events between the T and R formulations.

**TABLE 4:** Adverse events after administration of test and reference formulations under fasted conditions.

	T Formulation (N = 18)		R Formulation (N = 18)		Total (N = 36)	
	Case	Subjects [N (%)]	Case	Subjects [N (%)]	Case	Subjects [N (%)]
TEAEs	7	5 (13.89)	4	4 (11.11)	11	9 (25.00)
Various inspection	6	5 (13.89)	3	3 (8.33)	9	8 (22.22)
Elevated blood thyroid stimulating hormone	2	2 (5.56)	0	0 (0)	2	2 (5.56)
Elevated blood triglycerides	0	0 (0)	2	2 (5.56)	2	2 (5.56)
Neutrophil count decreased	2	2 (5.56)	0	0 (0)	2	2 (5.56)
Alanine aminotransferase was elevated	1	1 (2.78)	0	0 (0)	1	1 (2.78)
Hyperuricemia	1	1 (2.78)	0	0 (0)	1	1 (2.78)
Elevated free thyroxine	0	0 (0)	1	1 (2.78)	1	1 (2.78)
All kinds of neurological diseases	0	0 (0)	1	1 (2.78)	1	1 (2.78)
Giddy	0	0 (0)	1	1 (2.78)	1	1 (2.78)
Diseases of blood and lymphatic system	1	1 (2.78)	0	0 (0)	1	1 (2.78)
Anaemia	1	1 (2.78)	0	0 (0)	1	1 (2.78)

Abbreviation: TEAEs, treatment emergent adverse events; N, number of subjects; T, test formulation; R, reference formulation.

## 4 Discussion

The objective of this study was to evaluate the bioequivalence of the T formulation produced by Renhexidelong Pharmaceutical Co. Ltd. in comparison to Nexavar^®^ (0.2 g) sorafenib tosylate tablets manufactured in-house by Bayer AG, which served as references.This well-designed and executed bioequivalence trial provides new data for pharmacokinetic studies of sorafenib in the Chinese population and serves as a reference for future clinical trials.

The pharmacokinetic parameters of sorafenib exhibit substantial inter-individual variability. Under oral doses of 0.2 g or 0.4 g twice daily, the variability range (%CV) of sorafenib exposure was 5%–83%, while the variability in plasma peak concentrations ranged from 33% to 88% (Jain, L. et al., 2011). A Phase I clinical trial of sorafenib demonstrated that in fasted patients with advanced solid tumors following a single 0.2 g oral dose (N = 5), the geometric mean values for AUC, C_max_, and t_1/2_ were 31.9 mg h/L, 1.08 mg/L, and 29.5 h, respectively (Strumberg D et al., 2005). In contrast, the geometric means observed in this study were 24.5 mg h/L for AUC, 1.10 mg/L for C_max_, and 23.9 h for t_1/2_, indicating lower systemic exposure, comparable C_max_, and a shorter half-life compared to advanced solid tumor patients. In a study by Huh et al., healthy male volunteers under fasting conditions (N = 8) receiving a single 0.2 g sorafenib dose showed a median T_max_ of 4.0 h and t_1/2_ of 22.2 ± 5.1 h, consistent with our findings. However, our study revealed lower systemic exposure, which may be attributed to inter-individual variability and the limited sample size of the reference studies.

Enterohepatic circulation (EHC) refers to a physiological process whereby certain drugs excreted via biliary pathways into the intestinal lumen undergo reabsorption and re-entry into the hepatic-portal circulation. This recirculation mechanism results in prolonged systemic retention of the drug, manifesting as secondary plasma concentration peaks at delayed time intervals and an extended elimination half-life (Malik et al., 2016).The pharmacokinetic profile of sorafenib may be influenced by EHC, a phenomenon corroborated by clinical observations. Dual-peak characteristics in plasma concentration-time curves have been consistently reported in treated patients, suggesting EHC-mediated reabsorption (Jain et al., 2011). Consistent with these findings, the concentration-time profile observed in our study (Figure 2) exhibited irregular absorption patterns with substantial inter-individual variability. A primary peak occurred at 4 h post-dose, followed by secondary and tertiary concentration surges at 10 and 24 h, respectively. Furthermore, following oral administration of the T formulation, a longer elimination half-life of 25.8 h was observed, with a notably slow terminal elimination phase—a pharmacokinetic hallmark consistent with the characteristics of EHC.

High-variance drugs exhibit an intra-individual variability of ≥30% in pharmacokinetic parameters (C_max_ or AUC), and sorafenib, a tyrosine kinase inhibitor, falls under this category of drugs (Huh et al., 2021; Song et al., 2021; Karalis et al., 2012; Lennernäs et al., 2024). Evaluating bioequivalence of highly variable drugs is complex and challenging, often requiring an increase in the sample size to meet the bioequivalence acceptance criteria (Davit et al., 2012), which in turn increases the cost of human resources and inputs. Both the Food and Drug Administration (FDA) (Haidar et al., 2008a; Haidar et al., 2008b) recommend the use of full-replica or half-replica designs for bioequivalence analyses of highly variable drugs; specifically, the reference product should be administered at least twice per individual (Karalis et al., 2012; Haidar et al., 2008a; Haidar et al., 2008b). Considering this characteristic, this trial utilized a full replicated crossover study design and enrolled 36 participants. The intra-individual standard deviations (S_WR_) of C_max_, AUC_0-t_, and AUC_0- ∞_ for the T formulation in the bioequivalence analysis results of this trial were 0.40, 0.43, and 0.42, respectively, which all exceeded the threshold of 0.294, that is, the intra-individual variability of sorafenib was ≥30%, confirming its highly variable nature, which is consistent with the findings reported in the literature.

It has been shown that Sorafenib binds to plasma proteins at a rate of 99.5% and has similar bioavailability when consumed with a moderate-fat diet compared to fasting. However, when consumed with a high-fat diet, the absorption of sorafenib was reduced by 30% compared with that in the fasted state (Clark et al., 2005). To accurately reflect the pharmacokinetic process of absorption, distribution, and elimination of sorafenib toluenesulfonate *in vivo*, this trail was conducted in the fasted state.

A total of 11 adverse events were reported in 9 subjects during this study, where 7 events were classified as mild and 4 as moderate in severity. 3 cases were likely unrelated to the test drug, while the remaining 8 events could not be ruled out as connected to the test drug and were all deemed adverse reactions to the drug. Fortunately, all 11 adverse events resolved without any subsequent effects by the end of the trial. There were no serious adverse events or withdrawals due to adverse events during the oral T and R formulations phases, indicating that 0.2 g sorafenib tosylate tablets are safe and well-tolerated in healthy Chinese subjects.

This study has several limitations that should be acknowledged. First, the sample size, although meeting the minimum regulatory requirements for bioequivalence trials, was relatively small. This may limit the ability to fully characterize inter-individual variability in pharmacokinetic parameters and safety profiles. Second, the exclusion of special populations (e.g., adolescents, children, elderly individuals) precludes extrapolation of the current findings to these groups. Future studies are warranted to assess the safety and efficacy of the T formulation in populations with distinct metabolic or physiological conditions. Furthermore, the safety assessment was confined to adverse drug reactions observed within a short-term period (≤120 h post-dose). Notably, the characteristic toxicities of sorafenib, such as hand-foot syndrome and hypertension, are typically associated with cumulative exposure and emerge following chronic administration (Boudou-Rouquette et al., 2012). Consequently, the transient monitoring window in this single-dose study may underestimate the clinically relevant risks of the T formulation in real-world settings where prolonged therapeutic use is required.

## 5 Conclusion

This randomized, open, single-dose, four-period crossover trial confirmed that sorafenib tosylate tablets (0.2 g), manufactured by Renhexidelong Pharmaceuticals Co., Ltd., were bioequivalent to the control drug Nexavar^®^ (Bayer AG, Germany), and were safe and well tolerated in healthy adult Chinese subjects under fasting conditions.

## Data Availability

The original contributions presented in the study are included in the article/supplementary material, further inquiries can be directed to the corresponding authors.
